# Co-circulation of H5N6, H3N2, H3N8, and Emergence of Novel Reassortant H3N6 in a Local Community in Hunan Province in China

**DOI:** 10.1038/srep25549

**Published:** 2016-05-06

**Authors:** Xuyong Li, Jiayun Yang, Bin Liu, Yane Jia, Jing Guo, Xue Gao, Shaoting Weng, Maijuan Yang, Liang Wang, Lin-Fa Wang, Jie Cui, Hualan Chen, Qiyun Zhu

**Affiliations:** 1 State Key Laboratory of Veterinary Etiological Biology, Lanzhou Veterinary Research Institute, CAAS, 1 Xujiaping, Lanzhou 730046, China; 2 Key Laboratory of Special Pathogens and Biosafety, Center for Emerging Infectious Diseases, Wuhan Institute of Virology, Chinese Academy of Sciences, 430071, Wuhan, China; 3 Programme in Emerging Infectious Diseases, Duke-NUS Medical School, Singapore 169857; 4 State Key Laboratory of Veterinary Biotechnology, Harbin Veterinary Research Institute, CAAS, 427 Maduan Street, Harbin 150001, China

## Abstract

Multiple infections of avian influenza viruses (AIVs) in poultry or wild birds contribute to the continued evolution of H5 subtype viruses in nature and provide potential recombination of AIVs of different origins. In this study, we carried out surveillance of AIVs in ducks, geese and the environment of a community in Hunan province, China, from 2014–2015. We isolated multiple co-circulated AIVs including H3N2, H3N8, and H5N6, and, most importantly, a novel reassortant: H3N6. Phylogenetic analyses suggest that H3N6 is highly likely derived from H5N6, which has recently been shown to have zoonotic potential with human infections. Studies with mammalian cell lines and a mouse model indicate that four selected AIVs of duck or goose origin can infect MDCK and A549 cells but have low pathogenicity in mice. We propose that a potential co-circulation of multiple subtypes including H5N6 in local area may result in the production of novel subtypes such as H3N6 by gene reassortment.

Waterfowl such as wild birds, ducks, and geese are natural reservoirs of avian influenza viruses (AIVs) globally, and most highly pathogenic human influenza viruses originate from waterfowl^1^,^2^,^3^,^4^,^5^,^6^. At this time, 16 hammagglutinins (HAs) and 9 Neuraminidases (NAs) of AIVs have been isolated from aquatic birds and among the AIVs capable of infecting humans, H5 and H7 (N9) are the most commonly reported^7^,^8^,^9^,^10^,^11^. Importantly, asymptomatic infection of waterfowl with most AIVs can promote evolution of influenza viruses^12^. Domestic ducks and geese are positioned at the interface between wild aquatic birds and terrestrial poultry due to free-range farming in southern China. For these farms, no biosecurity measures are used and open feeding, and lack of no disinfection plus close animal contact between migratory birds and domestic ducks and geese who share water, food, and housing create problems. Thus, AIVs of various sources co-exist in the same ecosystem, facilitating interspecies transmission and viral gene reassortment. Co-infection with different AIVs in waterfowl is generally considered a significant mechanism for promoting viral diversity by generating novel reassortants^1^,^4^,^13^.

The highly pathogenic H5N1 virus was first identified in sick geese in Guangdong Province in 1996^14^. In 1997, H5N1 reassortants, with the HA gene derived from A/goose/Guangdong/1/96-like viruses, caused lethal outbreaks in poultry and humans in Hongkong^15^. Since the beginning of 2004, significant outbreaks of H5N1 viral infection in poultry farms caused millions of domestic poultry deaths^3^,^6^,^16^. In 2005, thousands of migratory aquatic birds died in the Qinghai Lake area due to H5N1 viral infection^3^,^6^. Then, the Qinghai Lake H5N1 virus spread to Europe and Africa and exacerbated the global prevalence of the virus. Recently, a novel H5N6 virus was isolated in Asia, which was a reassortant of H5N1 and H6N6 viruses^17^,^18^,^19^. Such a novel virus had high pathogenicity in chickens and led to several human infections since 2014 in China^20^,^21^. Moreover, the H5N6 virus has also been isolated from domestic cats and wild birds in China^22^, suggesting that mammals and wild birds may contribute to viral circulation.

The H3 influenza virus is widely circulated in various species and has been frequently isolated from human, swine, birds, or wild animals. H3 subtypes have established lineages in domestic poultry and have caused mild or severe disease and multiple subtypes such as H3N2, H3N6 and H3N8 viruses have been isolated from domestic poultry globally^23^,^24^,^25^,^26^,^27^,^28^,^29^. Experimental studies showed that avian-origin H3 influenza viruses can replicate in the respiratory tract of mice, implying that H3 isolates from birds can have a potential risk to public health^30^,^31^.

Co-circulation of AIVs involving H3 and H5 subtypes remains poorly understood. In the winter of 2014, we targeted farms in Wugang city, Hunan Province, China and collected specimens from domestic ducks and geese and from the water of the duck farms. In this study, we report novel reassortants of H3 and H5 influenza viruses originated from a common ancestor in domestic ducks and highlight the need for control of H5N6 viral infection in waterfowl from poultry farms.

## Results

### Sample collection and virus isolation

Two samplings were conducted from November of 2014 to April of 2015 in our long-term surveillance project. Sample collection sites were along a river in Wugang city, Hunan Province, China. Domestic duck and goose farming in this region has grown substantially in recent years. Most farms were built in high-density areas lacking biosecurity measures. For some farms, healthy and sick ducks or geese were housed together. In all, 86 influenza strains from 160 samples from sick birds (positive rate 53.8%) were successfully isolated (unpublished data). No virus was isolated from fecal samples and 10 strains were selected for phylogenetic analysis and biological experiments (Table 1).

### Phylogeny and detection of novel reassortment

**Figure 1:**
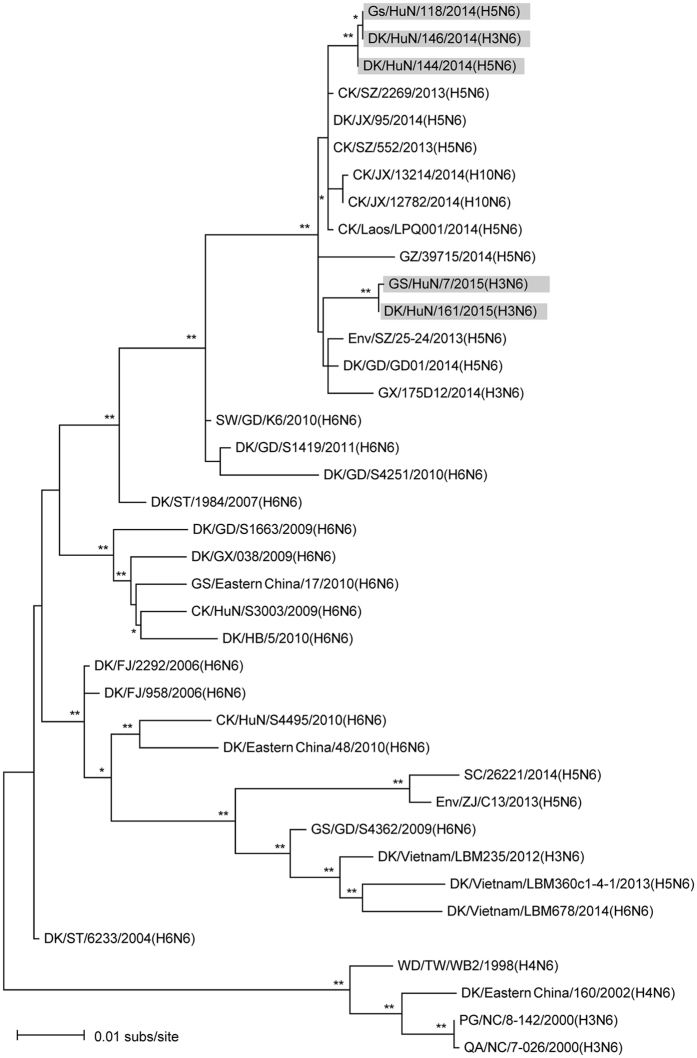
Phylogenetic relationships of N6 viruses. Phylogenic tree of NA genes of the N6 is mid-point rooted for clarification only. Bootstrap values less than 70% are not shown, and two-star (**) represents high values (> 90%) and one-star (*) represents medium (> 70% < 90%). The bar represents nucleotide substitutions per site (subs/site). Viruses included in this study are gray. Abbreviations are as follows: BG, bean goose; CK, chicken; DK, duck; Env, environment; Gs, goose; Md, Mallard; MD, Muscovy duck; PG, Pigeon; QA, quail; SW, swine; WD, wild duck; AH, Anhui; FJ, Fujian; GD, Guangdong; GY, Guiyang; GX, Guangxi; GZ, Guizhou; HuB, Hubei; HK, Hongkong; HuN, Hunan; JS, Jiangsu; JX, Jiangxi; SC, Sichuan; SD, Shandong; SX, Shanxi; ST, Shantou; SZ, Shenzhen; XJ, Xinjiang; YN, Yunnan; ZJ, Zhejiang.

**Figure 2:**
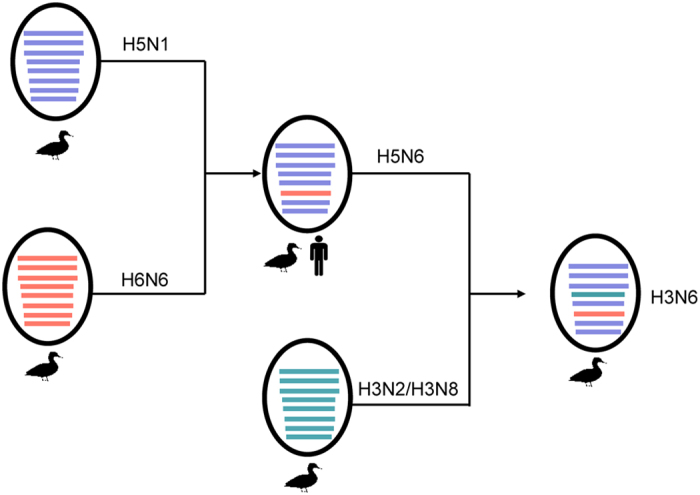
Possible genesis of the novel H3N6 reassortant. Illustration of original reassortment event of H5N1 and H6N6 viruses that generated the novel H5N6; and the subsequent reassortment between H5N6 and H3N2 or H3N8 viruses, which led to generation of the novel H3N6.

To investigate the genetic relationship between these co-circulated AIVs, we first compared the sequences of both key viral genes. HA genes of the four H3N2, three H3N6 and one H3N8 viruses shared 87.8–100% nucleotide identity. The HA gene of DK/HuN/146/2014 (H3N6) shared 87.8–91.2% identity at the nucleotide level with the four H3N2 viruses. The nucleotide identity of the HA gene of DK/HuN/199/2014 (H3N8) and the four H3N2 viruses was only 88.1–92.3% identical and was the most similar to A/aquatic bird/Korea/CN-1/2004 (H3N6) (94%). The nucleotide identity of HA for the four H3N2 viruses was 88.2–100%. Phylogenetic analysis of the H3 data sets showed that N2, N6, and N8 were commonly reassorted in the local area (Fig. S1). NA genes of the four H3N2 viruses shared 93–99.1% nucleotide identity and clustered into an Eurasian lineage of avian-like H3N2 viruses. The NA gene of A/duck/Hunan/199/2014 (H3N8) had the most similarity with A/chicken/Vietnam/G14/2008 (H3N8) (97%). N2 viruses isolated in our study were of two genetically distinct groups, suggesting multiple introductions and co-infection at local areas. The three N6 isolates, one H3 and two H5, were clustered closely with supported high bootstrap values (Fig. 1; Fig. S1A), indicating a possible H5N6 origin of the novel reassortant H3N6 virus (Fig. 2). At the time of manuscript preparation, a H3N6 virus—A/duck/Guangxi/175D12/2014—isolated from a native duck in Guangxi Province in 2014, was reported^32^. This sequence was added to our analysis and it was phylogenetically close to H5N6 (Fig. 1), but there was no direct relationship (i.e. high bootstrap supported). Due to the different geographic locations of such H3N6 virus and more isolates (three strains isolated in 2014 and 2015), possibly circulation of the H3N6 virus in ducks of southern China has been underestimated.

The HA of Gs/HuN/118/2014 (H5N6) and DK/HuN/144/2014 (H5N6) viruses shared 99.3% similarity at the nucleotide level and 97.9% identity with the human virus A/Guangzhou/39715/2014 (H5N6). Phylogenetic analysis showed that the two H5N6 viruses were grouped into clade 2.3.4.4 with other novel avian and human H5N6 viruses isolated in Asia (data not shown). The two H5N6 viruses were grouped into clade 2.3.4.4 with another novel avian and human H5N6 viruses isolated in Laos and southern China after 2013^33^,^34^, supporting their recent origin.

### Molecular characteristics

Molecular analysis revealed the presence of the PEKQTR/GLF motif of a single basic amino acid (aa) (Arginine) residues present at the cleavage site of HA protein of the newly isolated H3N6 and H3N2 and H3N8, which was characteristic of low pathogenic AIVs (LPAIV). Such a motif was different from the RERRRKR/GLF of HA of the two H5N6 viruses, representing high pathogenicity in chickens (Table 2). Several amino acid changes in HA, including T160A, Q226L and G228S, was reported to promote an affinity of AIVs for human-type receptors^35^,^36^. Analysis of the receptor-binding site revealed characteristics of an avian-like receptor binding motif present at the position of 226 and 228 in HA. However, the mutation of 160A was detected in two strains of H3N2. The NA deletion (amino acid position 59–69) was present in H3N6 and H5N6 viruses, but not in H3N2 and H3N8 viruses. The amino acid at position 591, 627 and 701 in PB2 suggested that these viruses are low pathogenic in mice^37^,^38^,^39^, however, mutations in M1 and NS1 amino acids indicated that these viruses may have acquired replication ability in mammals^40^,^41^. Thus, the H3N6 virus isolated in ducks possessed a unique characteristic compared with H3N2 and H3N8 viruses and shared some molecular characteristics with the H5N6 virus.

### Replication of H3N2, H3N6, H3N8, and H5N6 viruses in mammalian cells

**Figure 3:**
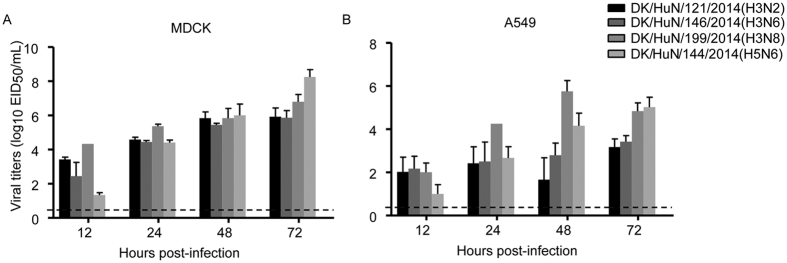
Replication curves of H3N2, H3N6, H3N8, and H5N6 viruses in mammalian cells. MDCK and A549 cells were inoculated with the virus (10^4^ EID_50_) and supernatants were collected for titration. (**A**) Viral growth curves in MDCK cells. (**B**) Viral growth curves in A549 cells. Data shown are viral titers (log_10_ EID_50_/mL) and the dashed lines indicate lower limits of detection.

One isolate each from H3N2, H3N6, H3N8, and H5N6 was selected for evaluating replication in mammalian cells. In MDCK cells, the replication of three H3 influenza viruses was markedly higher than that of H5N6 at 12 h after infection; however, viral titers were lower than that of DK/HuN/144/2014 (H5N6) at 72 h post infection (Fig. 3A). Three H3 influenza viruses (DK/HuN/121/2014, DK/HuN/146/2014 and DK/HuN/199/2014) replicated efficiently in human A549 cells. DK/HuN/144/2014 (H5N6) replicated well in human A549 cell after 24 h post infection. These results indicated that selected H3 strains and H5N6 virus from ducks were capable of efficient replication in mammalian cells.

### Virulence of H3N2, H3N6, H3N8 and H5N6 viruses in mice

**Figure 4:**
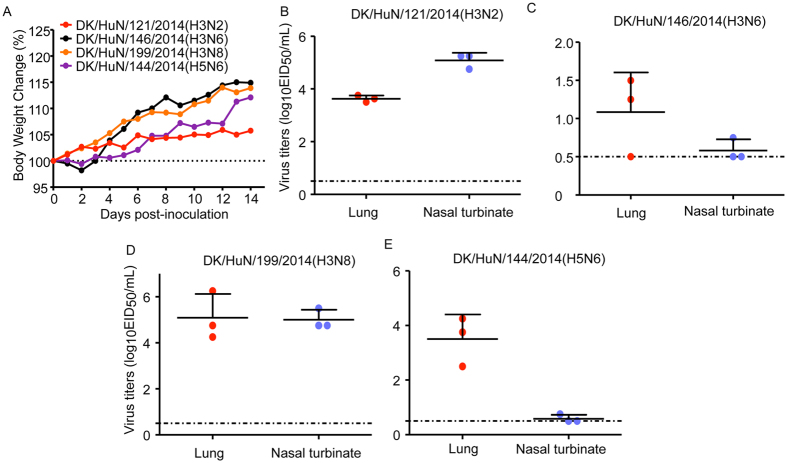
Virulence and replication of H3N2, H3N6, H3N8, and H5N6 viruses in mice. (**A**) Virulence of H3N2, H3N6, H3N8, and H5N6 viruses in mice. Mice were inoculated with H3N2, H3N6, H3N8, and H5N6 viruses (10^6^ EID_50_) in 50 μ L. Mouse weight was monitored daily for two weeks. (**B–E**) Replication of H3N2, H3N6, H3N8, and H5N6 viruses in mice. Three mice were euthanized on days 3 post-inoculation, and the nasal turbinates, lungs, kidneys, spleens, and brains were collected for viral titration in chicken eggs. Data shown are viral titers (log_10_ EID_50_/mL) from three mice; error bars indicate standard deviations.

Four influenza viruses tested in mammalian cells were evaluated for pathogenicity in mice. After inoculation of six-week-old BALB/c mice with 10^6^ EID_50_ of each virus, no disease or death occurred during the two-week observation period (Fig. 4A). DK/HuN/121/2014 (H3N2) and DK/HuN/199/2014(H3N8) replicated to high titers not only in lungs, but also in the nasal turbinates (Fig. 4B,D). Although DK/HuN/146/2014(H3N6) and DK/HuN/144/2014(H5N6) replicated efficiently in lungs, they barely replicated in nasal turbinates (Fig. 4C,E), suggesting that the gene recombination of these two strains might not be suited for efficient replication in mammals prior to forming a stable lineage. No virus was detected in brain, spleen, or kidney. Thus, AIVs originating from ducks or geese caused infection in mice, but no obvious disease or lethality.

## Discussion

Hunan Province in China is on the Eastern Asian-Australian Flyway of migratory birds. Duck and goose farms in this region are poorly built and domestic duck and goose farming occurs in high-density settings and in a free-range manner with no biosecurity measures, making this region an excellent ecosystem for AIVs cohabitation from domestic or migratory birds. In addition, ducks or geese from some poultry farms were imported and subsequently sold in live-poultry markets, which offer additional opportunities for virus reassortment and evolution with foreign AIVs circulating outside of Hunan Province. Therefore, this pattern of breeding, farming, and marketing has led to the co-existence of influenza viruses of different sources in farms, and the facilitation of interspecies transmission and viral gene reassortment. To prevent and control epidemic viral distribution, measures including closed feeding, free-transportation ban and active surveillance should be strictly implemented.

Our phylogenetic analyses demonstrated that reassortment frequently occurred in H3 and N6 subtypes in local duck and goose farms. Previous work indicated that H5N6 viruses were novel reassortants that emerged in Southeast Asia, with N6 derived from H6N6^18^,^20^,^22^,^42^. Phylogenetic analyses showed that all NA, PB2, PB1, PA, NP, M, and NS of the novel reassortant H3N6 were derived from the recently emerged H5N6 (Fig. 1 and Fig. S1), while its HA gene was from H3N2 or H3N8 isolated from southern China. Meanwhile, we successfully isolated H3N2, H3N8, and H5N6 viruses from ducks and geese at the same location, implying that co-circulation of multiple subtypes facilitates influenza viral evolution and reassortment in aquatic birds.

Waterfowl are chief natural hosts of H3N2, H3N6, H3N8, and H5N6 viruses. In the literature, we confirmed that most H3N2, H3N8, and H5N6 viruses reported from 2012 to 2014 were isolated from waterfowl, especially mallard and Muscovy ducks, and few were isolated from chickens. These results suggested that waterfowl, but not domestic chickens, contributed to the evolution of H3N2, H3N8, and H5N6 viruses. Human infections with H5N6 virus in China underscore a potential public health risk of the virus. In our study, the two H5N6 isolates had low mammalian pathogenicity, despite efficient replication in mammalian cells. Similarly, the novel H3N6 reassortant also had low virulence in mice. Thus, H3N6 and H5N6 AIVs have not yet fully adapted and may cause severe diseases in mammals if allowed to evolve further.

In summary, our study demonstrates that domestic duck and goose farms in southern China provide an ecosystem for co-circulation of multiple AIVs and facilitate the generation of novel reassortants such as H3N6 that could potentially infect mammals. Thus, large-scale surveillance of related AIVs in waterfowl in farms and mammals in surrounding areas are urgently needed in southern China.

## Methods

### Facility and ethics statement

All experiments with live H5N6 viruses were conducted within the enhanced animal biosafety level 3+ (ABSL3+ ) facilities in the Lanzhou Veterinary Research Institute (LVRI) and Harbin Veterinary Research Institute (HVRI) of the Chinese Academy of Agricultural Sciences (CAAS). This study was carried out in strict accordance with the recommendations in the Guide for the Care and Use of Laboratory Animals of the Ministry of Science and Technology of the People’s Republic of China. The protocols for animal studies were approved by the Committee on the Ethics of Animal Experiments of both LVRI and HVRI.

### Virus isolation

A total of 160 samples were collected from eight poultry farms along a river from ducks and geese. In addition, 10 fecal samples and 10 water samples were also collected. Each tissue, swab, fecal or water sample was placed in 2 mL of minimal essential medium supplemented with penicillin (2000 U/mL) and streptomycin (2000 U/mL). The samples were immediately frozen with dry ice and kept on dry ice during transfer to the laboratory. All of the individual samples were inoculated into 10-day-old embryonated chicken eggs for 48 h at 37 °C. The allantoic fluid samples were collected and tested for HA activity with 1% chicken red blood cells. All the positive ones were aliquoted and stored at − 80 °C. To avoid cross-contamination during egg inoculation and allantoic fluid collection, strict sterile techniques were implemented. All of the instruments used during the procedure were autoclaved before use and disinfected with 70% ethanol after use with each egg. Each specimen was divided into two aliquots and only one aliquot was used for the virus isolation procedure. Where HA assay was positive, hemagglutinin inhibition (HI) assay was performed to determine the HA subtype of the isolated AIV and Newcastle disease virus (NDV). Hemagglutinin (HA) subtypes were first determined by chicken serum anti-HA of each subtypes, then specific PCR methods was used to further identify HA and NA subtypes.

### Viruses and cells

The H3N2, H3N6, H3N8 and H5N6 viruses used in this study were isolated by using 10-day-old specific pathogen-free embryonated chicken eggs. The viruses were stocked in − 70 °C until used. Madin–Darby canine kidney (MDCK) cells were grown in Dulbecco’s modified Eagle’s medium (Gibco) supplemented with 5% fetal bovine serum plus with 100 UI/mL penicillin and 100 μ g/mL streptomycin. A549 cells were maintained in F-12K Nutrient Mixture (Gibco) supplemented with 10% fetal bovine serum plus with 100 UI/mL penicillin and 100 μ g/mL streptomycin. All cells were incubated at 37 °C with 5% CO_2_.

### RNA extraction and RT-PCR amplification

Viral RNAs were extracted from virus-infected allantoic fluid with the viral RNA mini kit (QIAGEN). cDNAs were synthesized from vRNAs by reverse transcription with Uni12 primer, and amplified by PCR with primers complementary to the conserved promoter and non-coding region of each gene segment (primers sequences were available on request).

### Evolutionary analysis

Phylogenetic relationships were inferred using the maximum likelihood (ML) method available in PhyML (version 3.1)^43^ and verified in MEGA (version 5.2.2)^44^ using the same method. SPR (subtree pruning and regrafting) branch-swapping and 1,000 bootstrap replications were used to determine the robustness of each node. The jModelTest (version 2.1.7)^45^ was used to select the best-fit model of nucleotide substitution, which are: GTR+ I for N6 data set, K2+ Γ for M1, HKY+ Γ for NP, GTR+ Γ for PA, GTR+ Γ for PB1, and GTR+ Γ for PB2. All sequences were aligned in MUSCLE (version 3.8.31)^46^, guided by the aligned protein sequences.

### Viral growth curves in cells

Viruses were inoculated into MDCK and A549 monolayers with 10^4^ 50% egg-infectious-dose (EID_50_). One hour after infection, the cells were replaced with fresh OPTI-MEM (containing 0.25 μ g/mL TPCK-trypsin for H3 subtype viruses) and incubated at 37 °C. Virus-containing culture supernatants were collected at various time points, hours post-infection (h.p.i), and titrated in eggs. The growth data shown was the average of three independent experiments.

### Mouse study

To evaluate virulence and replication of AIVs in mammals, five groups of BALB/c mice (6 weeks old female from Vital River Co. Ltd., Beijing, China) were inoculated with viruses at 10^6^ EID_50_ in a volume of 50 μ L. To determine the replication of the viruses in mice, three mice were euthanized at 3 days post-inoculation (d.p.i), their nasal turbinates, lungs, kidneys, spleens, and brains were collected for virus titration in eggs. The rest of the mice were observed for disease and death for two weeks.

## Additional Information

**How to cite this article**: Li, X. *et al.* Co-circulation of H5N6, H3N2, H3N8, and Emergence of Novel Reassortant H3N6 in a Local Community in Hunan Province in China. *Sci. Rep.* **6**, 25549; doi: 10.1038/srep25549 (2016).

## Figures and Tables

**Table 1:** H5N6, H3N2, H3N8, and H3N6 isolated from ducks, geese and environment during 2014–2015, China.

Viruses	Abbreviations	Isolation locations	Collection date	Host	GeneBank Accession nos.
A/goose/Hunan/118/2014 (H5N6)	Gs/HuN/118/2014(H5N6)	Wugang, Hunan, China	Nov 12, 2014	Goose	KX121195-121202
A/duck/Hunan/144/2014 (H5N6)	DK/HuN/144/2014(H5N6)	Wugang, Hunan, China	Nov 13, 2014	Duck	KX121203-121210
A/duck/Hunan/121/2014 (H3N2)	DK/HuN/121/2014(H3N2)	Wugang, Hunan, China	Nov 12, 2014	Duck	KX121211-121218
A/duck/Hunan/122/2014 (H3N2)	DK/HuN/122/2014(H3N2)	Wugang, Hunan, China	Nov 12, 2014	Duck	KX121219-121226
A/duck/Hunan/139/2014 (H3N2)	DK/HuN/139/2014(H3N2)	Wugang, Hunan, China	Nov 12, 2014	Duck	KX121227-121234
A/water/Hunan/140/2014 (H3N2)	Env/HuN/140/2014(H3N2)	Wugang, Hunan, China	Nov 12, 2014	Environment	KX121235-121242
A/duck/Hunan/199/2014 (H3N8)	DK/HuN/199/2014(H3N8)	Wugang, Hunan, China	Nov 14, 2014	Duck	KX121243-121250
A/duck/Hunan/146/2014 (H3N6)	DK/HuN/146/2014(H3N6)	Wugang, Hunan, China	Nov 13, 2014	Duck	KX121251-121258
A/duck/Hunan/161/2015(H3N6)	DK/HuN/161/2015(H3N6)	Wugang, Hunan province, China	April 6, 2015	Duck	KX121259-121266
A/goose/Hunan/7/2015 (H3N6)	Gs/HuN/7/2015(H3N6)	Wugang, Hunan, China	April 6, 2015	Goose	KX121267-121274

**Table 2:** Molecular characteristics of H3N2, H3N6, H3N8, and H5N6.

Gene segment	Amino acids mutations	Viruses used in this study			
		H3N2	H3N6	H3N8	H5N6
HA					
HA cleavage site		PEKQTR/GLF	PEKQTR/GLF	PEKQTR/GLF	RERRRKR/GLF
Increased transmission in guinea pigs	T160A	A/S	A	A	A
Increased affinity to human-type receptor	Q226L	Q	Q	Q	Q
	G228S	G	G	G	G
NA					
59–69 deletion		No	Yes	No	Yes
PB2					
Enhanced replication efficiency and increased virulence in mice	Q591K	Q	Q	Q	Q
Increased virulence in mice	E627K	E	E	E	E
Increased virulence in mice and transmission in mammals	D701N	D	D	D	D
NP					
Enhanced replication efficiency	N319K	N	N	N	N
M1					
Increased virulence in mice	N30D	D	D	D	D
	T215A	A	A	A	A
M2					
Amantadine resistance	S31N	S	S	S	S
NS1					
Increased virulence in mice	P42S	S	S	S	S
80–84 deletion		No	Yes	No	Yes
PDZ Motif		ESEV	ESEV	ESEV	ESEV

## References

[b1] Deng G. *et al.* Complex reassortment of multiple subtypes of avian influenza viruses in domestic ducks at the Dongting Lake Region of China. J Virol 87, 9452–9462 (2013). 23804642 10.1128/JVI.00776-13 PMC3754128

[b2] Chen H. *et al.* The evolution of H5N1 influenza viruses in ducks in southern China. Proc Natl Acad Sci USA 101, 10452–10457 (2004). 15235128 10.1073/pnas.0403212101 PMC478602

[b3] Chen H. *et al.* Properties and dissemination of H5N1 viruses isolated during an influenza outbreak in migratory waterfowl in western China. J Virol 80, 5976–5983 (2006). 16731936 10.1128/JVI.00110-06 PMC1472608

[b4] Dugan V. G. *et al.* The evolutionary genetics and emergence of avian influenza viruses in wild birds. PLoS Pathog 4, e1000076 (2008). 18516303 10.1371/journal.ppat.1000076 PMC2387073

[b5] Krauss S. *et al.* Influenza in migratory birds and evidence of limited intercontinental virus exchange. PLoS Pathog 3, e167 (2007). 17997603 10.1371/journal.ppat.0030167 PMC2065878

[b6] Liu J. *et al.* Highly pathogenic H5N1 influenza virus infection in migratory birds. Science 309, 1206 (2005). 16000410 10.1126/science.1115273

[b7] Webster R. G., Bean W. J., Gorman O. T., Chambers T. M. & Kawaoka Y. Evolution and ecology of influenza A viruses. Microbiol Rev 56, 152–179 (1992). 1579108 10.1128/mr.56.1.152-179.1992 PMC372859

[b8] Fouchier R. A. *et al.* Characterization of a novel influenza A virus hemagglutinin subtype (H16) obtained from black-headed gulls. J Virol 79, 2814–2822 (2005). 15709000 10.1128/JVI.79.5.2814-2822.2005 PMC548452

[b9] Easterday B. C., Trainer D. O., Tumova B. & Pereira H. G. Evidence of infection with influenza viruses in migratory waterfowl. Nature 219, 523–524 (1968). 4299258 10.1038/219523a0

[b10] Olsen B. *et al.* Global patterns of influenza a virus in wild birds. Science 312, 384–388 (2006). 16627734 10.1126/science.1122438

[b11] Munster V. J. *et al.* Spatial, temporal, and species variation in prevalence of influenza A viruses in wild migratory birds. PLoS Pathog 3, e61 (2007). 17500589 10.1371/journal.ppat.0030061 PMC1876497

[b12] Yoon S. W., Webby R. J. & Webster R. G. Evolution and Ecology of Influenza A Viruses. Curr Top Microbiol 385, 359–375 (2014). 10.1007/82_2014_396 24990620

[b13] Sharp G. B. *et al.* Coinfection of wild ducks by influenza A viruses: distribution patterns and biological significance. J Virol 71, 6128–6135 (1997). 9223507 10.1128/jvi.71.8.6128-6135.1997 PMC191873

[b14] Li Z. *et al.* The NS1 gene contributes to the virulence of H5N1 avian influenza viruses. J Virol 80, 11115–11123 (2006). 16971424 10.1128/JVI.00993-06 PMC1642184

[b15] de Jong J. C., Claas E. C., Osterhaus A. D., Webster R. G. & Lim W. L. A pandemic warning? Nature 389, 554 (1997). 9335492 10.1038/39218 PMC7095477

[b16] Chen H. H5N1 avian influenza in China. Sci China C Life Sci 52, 419–427, (2009). 19471864 10.1007/s11427-009-0068-6

[b17] Wong F. Y. *et al.* Reassortant highly pathogenic influenza A(H5N6) virus in Laos. Emerg Infect Dis 21, 511–516 (2015). 25695754 10.3201/eid2103.141488 PMC4344285

[b18] Wu H. *et al.* Novel reassortant highly pathogenic H5N6 avian influenza viruses in poultry in China. Infect Genet Evol 31, 64–67 (2015). 25653129 10.1016/j.meegid.2015.01.019

[b19] Li X. *et al.* Genetic and biological characterization of two novel reassortant H5N6 swine influenza viruses in mice and chickens. Infect Genet Evol 36, 462–466 (2015). 26296602 10.1016/j.meegid.2015.08.017

[b20] Yang Z. F., Mok C. K., Peiris J. S. & Zhong N. S. Human infection with a novel avian influenza A(H5N6) virus. N Engl J Med 373, 487–489 (2015). 26222578 10.1056/NEJMc1502983

[b21] World Health Organization. Global Alert and Response (GAR). http://www.who.int/csr/don/archive/country/chn/en/(26/01/2016) (2016).

[b22] Yu Z. *et al.* Fatal H5N6 Avian Influenza Virus Infection in a Domestic Cat and Wild Birds in China. Sci Rep 5, 10704 (2015). 26034886 10.1038/srep10704 PMC4603707

[b23] Campitelli L. *et al.* H3N2 influenza viruses from domestic chickens in Italy: an increasing role for chickens in the ecology of influenza? J Gen Virol 83, 413–420 (2002). 11807234 10.1099/0022-1317-83-2-413

[b24] Pu J. *et al.* Genetic analysis of H3 subtype influenza viruses isolated from domestic ducks in northern China during 2004–2005. Virus Genes 38, 136–142 (2009). 19067150 10.1007/s11262-008-0300-7

[b25] Zhou H. B., Zhang A. D., Chen H. C. & Jin M. L. Emergence of novel reassortant H3N2 influenza viruses among ducks in China. Arch Virol 156, 1045–1048 (2011). 21318308 10.1007/s00705-011-0940-0

[b26] Li Q. *et al.* Genome Sequence of a Novel Reassortant H3N6 Avian Influenza Virus from Domestic Mallard Ducks in Eastern China. Genome Announc 1, e0022312 (2013). 23580714 10.1128/genomeA.00223-12 PMC3624688

[b27] Brown J. D. *et al.* Intestinal excretion of a wild bird-origin H3N8 low pathogenic avian influenza virus in mallards (*Anas Platyrhynchos*). J Wildl Dis 48, 991–998 (2005). 23060500 10.7589/2011-09-280 PMC11373667

[b28] Choi J. G. *et al.* Genetic relationship of H3 subtype avian influenza viruses isolated from domestic ducks and wild birds in Korea and their pathogenic potential in chickens and ducks. Vet Microbiol 155, 147–157 (2012). 21955449 10.1016/j.vetmic.2011.08.028

[b29] Bean W. J. *et al.* Evolution of the H3 influenza virus hemagglutinin from human and nonhuman hosts. J Virol 66, 1129–1138 (1992). 1731092 10.1128/jvi.66.2.1129-1138.1992 PMC240817

[b30] Driskell E. A., Jones C. A., Stallknecht D. E., Howerth E. W. & Tompkins S. M. Avian influenza virus isolates from wild birds replicate and cause disease in a mouse model of infection. Virology 399, 280–289 (2010). 20123144 10.1016/j.virol.2010.01.005

[b31] Baz M., *et al.* Replication and immunogenicity of swine, equine, and avian h3 subtype influenza viruses in mice and ferrets. J Virol 87, 6901–6910 (2013). 23576512 10.1128/JVI.03520-12 PMC3676140

[b32] Liu T. *et al.* Characterization of the Whole-Genome Sequence of an H3N6 Avian Influenza Virus, Isolated from a Domestic Duck in Guangxi, Southern China. Genome Announc 3, e01190–15 (2015). 26472834 10.1128/genomeA.01190-15 PMC4611686

[b33] Zhang G. *et al.* Identification of an H6N6 swine influenza virus in southern China. Infect Genet Evol 11, 1174–1177 (2011). 21382518 10.1016/j.meegid.2011.02.023 PMC3104128

[b34] Wang G. *et al.* H6 influenza viruses pose a potential threat to human health. J Virol 88, 3953–3964 (2014). 24501418 10.1128/JVI.03292-13 PMC3993743

[b35] Vines A. *et al.* The role of influenza A virus hemagglutinin residues 226 and 228 in receptor specificity and host range restriction. J Virol 72, 7626–7631 (1998). 9696865 10.1128/jvi.72.9.7626-7631.1998 PMC110023

[b36] Gao Y. *et al.* Identification of amino acids in HA and PB2 critical for the transmission of H5N1 avian influenza viruses in a mammalian host. PLoS Pathog 5, e1000709 (2009). 20041223 10.1371/journal.ppat.1000709 PMC2791199

[b37] Hatta M., Gao P., Halfmann P. & Kawaoka Y. Molecular basis for high virulence of Hong Kong H5N1 influenza A viruses. Science 293, 1840–1842 (2001). 11546875 10.1126/science.1062882

[b38] Li Z. *et al.* Molecular basis of replication of duck H5N1 influenza viruses in a mammalian mouse model. J Virol 79, 12058–12064 (2005). 16140781 10.1128/JVI.79.18.12058-12064.2005 PMC1212590

[b39] Li X. *et al.* Genetics, receptor binding property, and transmissibility in mammals of naturally isolated H9N2 Avian Influenza viruses. PLoS Pathog 10, e1004508 (2014). 25411973 10.1371/journal.ppat.1004508 PMC4239090

[b40] Fan S. *et al.* Two amino acid residues in the matrix protein M1 contribute to the virulence difference of H5N1 avian influenza viruses in mice. Virology 384, 28–32 (2009). 19117585 10.1016/j.virol.2008.11.044

[b41] Jiao P. *et al.* A single-amino-acid substitution in the NS1 protein changes the pathogenicity of H5N1 avian influenza viruses in mice. J Virol 82, 1146–1154 (2008). 18032512 10.1128/JVI.01698-07 PMC2224464

[b42] Bi Y. *et al.* Two Novel Reassortants of Avian Influenza A (H5N6) Virus in China. J Gen Virol 96, 975–981 (2015). 25604926 10.1099/vir.0.000056

[b43] Guindon S. *et al.* New algorithms and methods to estimate maximum-likelihood phylogenies: assessing the performance of PhyML 3.0. Syst Biol 59, 307–321 (2010). 20525638 10.1093/sysbio/syq010

[b44] Tamura K., Peterson D., Peterson N., Stecher G., Nei M. & Kumar S. MEGA5: molecular evolutionary genetics analysis using maximum likelihood, evolutionary distance, and maximum parsimony methods. Mol Biol Evol 28, 2731–2739 (2011). 21546353 10.1093/molbev/msr121 PMC3203626

[b45] Darriba D., Taboada G. L., Doallo R. & Posada D. jModelTest 2: more models, new heuristics and parallel computing. Nat Methods 9, 772. 22847109 10.1038/nmeth.2109 PMC4594756

[b46] Edgar R. C. MUSCLE: multiple sequence alignment with high accuracy and high throughput. Nucleic Acids Res 32, 1792–1797. 15034147 10.1093/nar/gkh340 PMC390337

